# The impact of leadership on employee well-being: on-site compared to working from home

**DOI:** 10.1186/s12889-022-14612-9

**Published:** 2022-11-23

**Authors:** Daniel Lundqvist, Cathrine Reineholm, Christian Ståhl, Andreas Wallo

**Affiliations:** 1 Department of Behavioural Sciences and Learning, Linköping University, Linköping, SE-581 83 Sweden; 2 HELIX Competence Centre, Linköping University, Linköping, Sweden

**Keywords:** Manager, Supervisor, Subordinate, Health, Telework, Moderation

## Abstract

**Background:**

The Covid-19 pandemic has brought significant changes to the way people work and there are several reasons to believe that working from home will become more common in the future. Yet more knowledge is needed on whether the effectiveness of leadership differs if the work is performed remotely compared to on-site work.

**Purpose:**

The aim of this study is to examine the place of work as a moderator for the effectiveness of leadership on employee well-being.

**Method:**

A survey was answered by 364 white-collar workers, employed by a larger Swedish municipality, who because of the covid-19-pandemic were offered to work from home.

**Results:**

The employees working in their regular office perceived having more sufficient work equipment. No other differences were found in the investigated variables. Supportive leadership was associated with all investigated well-being variables in the hypothesised directions. Place of work did not moderate the relationship between Support leadership and the investigated well-being outcomes (Job satisfaction, Stress, General well-being).

**Conclusion:**

This study shows that there are few differences between employees working from home or working on-site during the Covid-19 pandemic. The supportive leadership of the closest manager seem to be important for well-being regardless of the worksite.

## Introduction

This paper addresses the topic of leadership in relation to employees’ well-being in remote work situations compared to work that is conducted on-site. The rationale behind the study is that while the leadership of managers has been shown to be important for employees’ well-being [1–9], research has not yet delved deeper into whether the effectiveness of leadership differs if the work is performed remotely compared to on-site work. The Covid-19 pandemic has brought significant changes to the way people work [10] and resulted in a rapid transition of working from home in several occupational groups [11]. Research has begun to investigate how the location of work impacts the effectiveness of leadership [12–17], but comparisons are often made between employees of different occupations, work and work tasks. Hence, research on the relative effectiveness of on-site and remote leadership in the same context is largely missing, which this study aims to provide. The Covid-19 pandemic and the increase of employees working from home, coupled with the Swedish pandemic strategy, provided the unique opportunity to study this issue in a homogenous sample of white-collar workers. These white-collar workers all worked in the same organization, had similar work tasks, and all had the opportunity to work from home, but some employees chose not to do so. Therefore, this study investigates whether the place of work is important for the relationship between supportive leadership and employee well-being. Supportive leadership is investigated as a broad theoretical construct encompassing leadership behaviours such as providing support, showing concern and empathy, which is found in several leadership theories related to employee wellbeing [1–8].

This study contributes to previous knowledge in several ways. Firstly, it includes a group that has not received a great deal of attention in previous research addressing the effects of Covid-19: those who decided to work in their regular office despite having the opportunity to work from home. Previous research has focused more on those working from home, or those who had to work from their regular office. Secondly, the study answers the call for more research on boundary conditions and the impact of situational conditions on the effect of leadership [4, 18]. More knowledge is needed on such conditions, and consequently, the aim of this study is to examine the place of work as a moderator for the effectiveness of leadership on employee well-being.

In the following section, we introduce the state of the current literature on leadership in relation to well-being and remote work, which leads up to the hypotheses tested in this particular study.

### Covid-19 pandemic and working from home

Unlike many other countries, Sweden chose not to introduce any hard lockdowns during the Covid-19 pandemic and in many Swedish organizations, work has been able to continue. In the spring of 2020, however, a recommendation was announced by the Public Health Agency of Sweden [19] that everyone who can, should work from home, which particularly public employers were expected to comply with. This recommendation was in place until 9^th^ of February 2022, except for a period in the fall of 2021. For many employees, this recommendation implied a rapid transition to working online with their homes as their physical work environment. While there are previous experiences of working from home in many occupational groups in Sweden, the pandemic and the governmental recommendations caused new and large groups to never or seldomly work from home to do so exclusively [11].

There are several reasons to believe that working from home will remain an option for many employees [20, 21]. A survey by Eurofund [22] showed that most people who worked at home full-time or part-time during the pandemic were positive about working from home also in the future. In many organizations, therefore, there is an ongoing discussion about how the work should be designed after the pandemic, often referred to as “work post-covid” or “the new normal” [20–24].

A key aspect of this discussion concerns managers and their leadership. One problem that managers raise with working from home during the covid-19 pandemic is the difficulty of maintaining good contact with their employees [23, 25–27]. It has been difficult to know how the employees are doing and how they enjoy their work when you cannot meet in person. At the same time, according to Swedish legislation [28] employers are obliged to provide a good work environment and a healthy workload. The rapid transition to work from home caused by Covid-19 provided little time for preparation and planning, and in many organisations, employees and not least managers were unprepared for how the work should be conducted and how the statutory obligations would be fulfilled. Because of the rapid increase in working from home, questions have arisen about managers’ leadership and how they should lead when the work is performed remotely.

### Leadership and well-being

The concept of leadership has been studied from different perspectives, and several theories exist [29, 30]. In this paper, leadership is defined as influencing employees to reach work-related goals [29], and an important part of this is arguably employees’ health and well-being. Well-being is an equally broad concept that encompasses several different dimensions [31–34], such as self-perceived health and well-being, but also the absence of negative experiences such as stress. Well-being can also be differentiated between general well-being and context-specific well-being (such as related to work) [35].

The importance of leadership for employee well-being has become an increasingly well-studied phenomenon over the past decade [18], and several literature reviews and meta-analyses have been conducted in this area [1–9]. They all show that the manager's leadership is associated with the employee's well-being. Specifically, leadership styles such as supportive leadership, relationship-oriented leadership and transformational leadership seem to be important for employees to feel satisfied with their jobs and experience well-being. Common for these styles, despite originating from different leadership theories, is that they all involve managers displaying behaviours of concern, support, and empathy.

Recently, studies conducted during the Covid-19 pandemic and the enforced work from home have shown that the same type of supportive leadership is central to employees' well-being. For example, studies show that a leadership style of the closest manager focused on the concern of the employees is related to increased job satisfaction [36, 37] and general well-being [38], decreased tension [39], stress and symptoms of burnout [15, 40, 41] of employees when working from home during the pandemic.

### Leadership and working from home

One issue that the debate on the new normal in working life has not addressed is whether the effectiveness of leadership differs if the work is performed remotely compared to on-site work, and whether leadership needs to be different. Several researchers have raised the need for more knowledge not only about whether there is an association between leadership and employee well-being, but also about when or for whom this association applies and, thus, also when or for whom it does not apply [4, 18]. Most leadership theories suggest that the most effective leadership is displayed when the manager and employee meet in person [42–44], but because of the Covid-19 pandemic and the sudden increase in working from home, this has not always been possible.

Previous research has shown both advantages and disadvantages of working from home [45–51]. Studies have shown that flexible work arrangements and opportunities to work from home improve employees’ health, instil a better work-life balance, and increase their sense of autonomy and self-leadership. Working from home may therefore increase the employee’s resources and diminish the importance of the manager’s leadership. However, other studies have found that working from home may have a detrimental effect on the well-being and health of employees, decrease the balance between work and private life, and generate feelings of loneliness and social isolation. As the social contacts and support from the workplace are remote, via digital platforms, it is a risk that employees feel excluded from the workplace and receive insufficient help and support. Studies have, however, found that managers compensated for the lack of personal contact at work during the Covid-19 pandemic by communicating more often with employees than before the pandemic to support them and to ensure the work went as planned [27, 52]. There seem to be two different ideas of how effective leadership becomes when employees work from home: increased resources resulting in leadership being less important, or, social isolation resulting in leadership being more important.

While there has been an increase in studies investigating the impact of leadership on employee well-being while working from home during the Covid-19 pandemic [15, 36–41], few of the studies have made comparisons with on-site workers. In a study conducted before the pandemic, Golden and Veiga [12] show that leadership is linked to employee well-being and that the amount of work from home moderates the relationship between leadership and well-being. There was a more pronounced relationship between leadership and well-being for those working from home to a high extent, compared to those who worked from home to a lesser extent. The authors conclude that those who worked from home to a high extent were more dependent on leadership to feel good than those who worked from home to a lesser extent. However, the study was done more than a decade ago. Much has happened in terms of technological development and tools, and above all, there was not a pandemic that encouraged people to work from home [53]. Those who worked from home in Golden and Veiga’s [12] study might have seen this as a form of reward or benefit, while this need not be the case for those working from home during the Covid-19 pandemic. Other studies, conducted during the pandemic, have performed separate analyses for employees working from home and for employees working on-site. Two studies found similar results regardless of the workplace, i.e., leadership was associated with employee well-being [13, 14], while two studies found the managers’ leadership was associated with the well-being of employees working from home, but not for the well-being of employees working on-site [16, 17]. One potential reason for these conflicting results may be related to the samples investigated, as these often are very heterogeneous and consists of different occupational groups, the groups have different work tasks and working from home was not offered to all. The amount of support needed from the manager is likely dependent on what kind of work and work tasks the employee is expected to perform. Furthermore, previous studies have shown that managers’ leadership may have different associations with different dimensions of well-being [7, 54]. For instance, only job satisfaction was used as a measure of well-being in the study by Golden and Veiga [12]. In order to get a more complete picture of the importance of leadership for employee well-being, well-being needs to be examined as a multidimensional concept [7]. To understand whether the place of work influence the effectiveness of leadership, the employee needs to be comparable, for instance regarding work tasks.

To summarize, previous research provides evidence that the supportive leadership of managers is associated with the well-being of employees. Regarding whether the effectiveness of leadership differs if the work is performed at home in contrast to when it is performed in the regular office, there seem to be two opposing ideas. One idea suggests that leadership is less effective for employees working from home as they gain more resources, such as autonomy and need to develop more self-leadership skills to maintain their work. The other idea suggests that leadership is more effective for employees working from home because of the reduced number of sources of support. While the empirical evidence is still conflicting, the current evidence suggests that leadership is equally or more important for employees working from home compared to employees working in the regular office [12–17].

Based on previous findings, the hypotheses for this study are as follows:

- *Hypothesis 1:* Supportive leadership is positively associated with employees’ Job satisfaction and General well-being, and negatively associated with employees’ Stress.
- *Hypothesis 2:* Place of work moderates the associations between the Supportive leadership in that the relationship is stronger for those working from home compared to those working from the office.

## Methods

### Sample and procedure

To investigate Hypothesis 2, whether the Place of work moderates the association between Supportive leadership and well-being, it is vital to make sure the result is not confounded by participants having different work tasks or demands of being present at the workplace. The researchers therefore contacted the Human Resource department of a larger Swedish municipality and explained the study, who agreed to participate. The Human Resource department identified different departments in the municipality where all employees had for the last 10–12 months been offered to work from home because of the Covid-19 pandemic. They also provided the researchers with the e-mail addresses of the employees. Thus, the sample of this study consists of white-collar workers employed by a larger Swedish municipality, who all had been offered to work from home. Working from home was not widely practiced before the pandemic in either of the investigated departments, according to the Human Resource department.

An electronic questionnaire was sent to the employees’ work e-mail in February 2021 (*N* = 790). After two reminders, the questionnaire had been answered by 364 (46%). Of these, 99 (27%) were men and 262 (73%) were women, their average age was 47.81 years (SD = 11.30). 296 (81%) had a university degree, and 51 (14%) had a secondary degree. Of the 364, 315 (87%) had been working from home while 49 (13%) had been working from their regular office on-site.

### Measures

#### Supportive leadership

Leadership was investigated using seven items from the third version of the Copenhagen Psychosocial Questionnaire (COPSOC) [55, 56]. An example item is: “How often do you get help and support from your immediate superior, if needed?”. Cronbach’s alpha for Supportive leadership was 0.92.

#### Place of work

Place of work was measured using a single question: “From where are you currently working?”. The response options were (0) mainly from my regular office, or (1) mainly from home.

#### Well-being

Three different measures were used to capture well-being, of which two were taken from the third version of COPSOC [55, 56]. The first scale, Job satisfaction, was measured using 4 items. An example item is: “How pleased are you with your job as a whole, everything taken into consideration?”. Cronbach’s alpha for Job satisfaction was 0.84. The second scale, Stress, consists of 3 items (Cronbach’s alpha = 0.81). An example item is: “How often have you been tense?”. General well-being was investigated using the Swedish version of WHO-5 [57] which consists of 5 items (Cronbach’s alpha = 0.87). An example item is: Over the last 2 weeks, I have felt cheerful and in good spirit”. The items of WHO-5 are answered on a 6-point Likert scale, ranging from “All the time (5)” to “At no time (0)”.

As all items from COPSOC were rated on a 5-point Likert-scale and coded as ranging between 0 and 100, the response range of WHO-5 was recoded to correspond to the 0–100 range of the other scales.

#### Confounders

Age, Gender, and the Level of education were treated as potential confounders. The perception of having suitable Work equipment (work desk, computer screens, etc.), and the frequency of Informal information and feedback meetings between the manager and employee were also considered potential confounders.

### Statistical analyses

A factor analysis was used to ensure construct validity of the scales. The Kaiser–Meyer–Olkin test result was 0.91, and Bartlett’s test of sphericity (*χ*^*2*^(170) = 4017.37, *p* < 0.001) showed that a factor analysis could be performed on the data. Based on eigenvalues greater than one, the factor analysis revealed a four-factor solution (Table 1) accounting for 69.70% of the variance. The variables were therefore considered distinct, and further analysis commenced.

**Table 1:** Rotated Component Matrix

	**Component**			
	**1**	**2**	**3**	**4**
Leadership 1	**0.80**	0.11	0.15	0.13
Leadership 2	**0.82**	0.11	0.13	0.18
Leadership 3	**0.69**	0.12	0.18	-0.04
Leadership 4	**0.81**	0.05	0.34	0.08
Leadership 5	**0.79**	0.09	0.25	0.12
Leadership 6	**0.79**	0.11	0.17	0.02
Leadership 7	**0.82**	0.09	0.21	0.07
Job satisfaction 1	0.33	0.18	**0.79**	0.08
Job satisfaction 2	0.19	0.13	**0.52**	0.29
Job satisfaction 3	0.34	0.08	**0.81**	0.10
Job satisfaction 4	0.32	0.18	**0.82**	0.18
Stress 1	-0.07	-0.33	-0.09	**-0.72**
Stress 2	-0.09	-0.19	-0.18	**-0.81**
Stress 3	-0.11	-0.27	-0.18	**-0.81**
Well-being 1	0.21	**0.72**	0.21	0.32
Well-being 2	0.16	**0.72**	0.14	0.44
Well-being 3	0.10	**0.83**	0.15	0.25
Well-being 4	0.02	**0.80**	0.03	0.15
Well-being 5	0.12	**0.74**	0.10	0.05

Bold numbers indicate the relevant factor each item belongs to

Descriptive statistics (mean values, and standard deviation) were calculated for the investigated variables. Their intercorrelation was analysed using Pearson correlation analysis (except for Level of education were Spearman’s rank-correlation was used).

Differences between the two workplaces were investigated using chi^2^ (Gender) and independent *t*-tests.

To investigate the moderation of Place of work on the association between Supportive leadership and the well-being outcomes, multiple linear regressions (method Enter) were performed for Supportive leadership in relation to each of the well-being outcomes. First, confounders (Age, Gender, Level of education, Work equipment, Informal meetings) were entered. Second, the independent variables, Supportive leadership and Place of work, were entered. In the last step, the interaction between Supportive leadership and Place of work was added to the model. The independent variables were grand mean centred.

All statistical analyses were performed using IBM SPSS 28.

## Results

Table 2 shows the mean and standard deviation of all investigated variables, and their intercorrelation. Supportive leadership was related to all the investigated outcome measures in the expected direction.

**Table 2:** Cronbach’s alpha, mean, and standard deviation of all variables and their intercorrelation (*N* = 364)

	*M*	*SD*	1	2	3	4	5	6	7	8	9
1.Age	47.81	11.30	1								
2.Gender^a^	0.72	0.45	-.06	1							
3.Education	2.88	0.45	-.05	.04	1						
4.Work equipment	74.24	21.63	.03	-.01	-.12*	1					
5.Meetings	75.78	22.47	.12*	.08	.06	.22**	1				
6.Workplace^b^	0.87	0.34	.01	.06	.06	-.11*	-.03	1			
7.Leadership	64.39	21.54	-.05	-.06	-.04	.28**	.45**	.01	1		
8.Job satisfaction	69.56	21.06	.07	-.04	-.08	.27**	.31**	-.07	.58**	1	
9.Stress	32.45	21.34	-.15**	.15**	.04	-.21**	-.19**	.01	-.27**	-.38**	1
10.Well-being	50.61	22.18	.04	-.12*	-.01	.20**	.28**	-.07	.28**	.38**	-.57**

^a^ Coded as 0 = Male, 1 = Female

^b^ Coded as 0 = Office, 1 = Home

^*^ *p* < .05

^**^ *p* < .01

To investigate if the two groups, those working from home (*n* = 315) and those working from their regular office (*n* = 49) differed regarding the investigated variables, chi-squared tests and *t*-tests were performed. No associations were found between Place of work and Gender (*χ*^*2*^(1) = 1.16, *p* = 0.281), or between Place of work and Level of education (*χ*^*2*^(2) = 5.12, *p* = 0.077). Furthermore, no differences were found between Place of work and the investigated variables (See Table 3), except that employees working from their regular office rated higher on access to relevant Work equipment (*t*(356) = 2.05, *p* = 0.041).

**Table 3:** Comparison between home workers and office workers

	Home (*n* = 315)	Office (*n* = 49)	*T* (df)
	*M* (*SD*)	*M* (*SD*)	
Age	47.78 (11.23)	47.70 (11.78)	-0.04 (357)
Work equipment	73.39 (21.57)	80.32 (21.45)	2.05 (356)*
Informal meetings	77.49 (22.43)	77.72 (23.11)	0.63 (353)
Supportive leadership	64.44 (21.64)	63.77 (21.28)	-0.20 (357)
Job satisfaction	69.57 (21.41)	73.14 (18.79)	1.26 (359)
Stress	32.32 (21.30)	32.09 (21.42)	-0.07 (356)
Well-being	50.17 (22.03)	54.62 (22.66)	1.18 (310)

^*^ *p* < .05

To investigate the two hypotheses, regression analyses were performed on Supportive leadership in relation to the different well-being outcomes (Table 4). Model 1 shows the association between Supportive leadership and Job satisfaction, Stress and Well-being, respectively, adjusted for Age, Gender, Work Equipment, and frequency of Informal meetings. Supportive leadership was significantly associated with all the investigated outcomes, i.e., Job satisfaction (*β* = 0.55, *t*(351) = 10.94, *p* < 0.001), Stress (*β* = -0.21, *t*(351) = -3.53, *p* < 0.001), and General well-being (*β* = 0.17, *t*(306) = 2.67, *p* = 0.008). Place of work was not significantly associated with any of the outcomes.

**Table 4:** Supportive leadership regressed on Job satisfaction, Stress, and Well-being, adjusted for Age, Gender, Level of Education, Work equipment, and Informal meetings, with Workplace as moderator (*N* = 364)

	Job satisfaction			Stress			Well-being		
	Model 1	Model 2	Model 3	Model 1	Model 2	Model 3	Model 1	Model 2	Model 3
	*β*	*β*	*β*	*β*	*β*	*β*	*β*	*β*	*β*
Age	0.03	0.09*	0.10	-0.12*	-0.14**	-0.14**	0.01	0.02	0.02
Gender	-0.06	-0.01	0.01	0.15**	0.13**	0.13*	-0.14*	-.012*	-.011*
Education	-0.08	-0.05	-.05	0.02	0.01	0.01	0.02	0.03	0.03
Work equipment	0.21***	0.11*	0.11*	-0.19***	-0.15**	-0.15**	0.15**	0.12*	0.12*
Meetings	0.26***	0.02	0.02	-0.15**	-0.06	-0.06	0.25***	0.18**	0.18**
Leadership		0.54***	0.54***		-0.20***	-0.20***		0.17**	0.17**
Workplace^a^		-0.06	-0.06		-0.02	-0.02		-0.05	-0.04
L x WP			0.04			-0.02			0.06
*R*^*2*^	0.15	0.37	0.37	0.11	0.14	0.14	0.12	0.14	0.14
*Adj R*^*2*^	0.14	0.35	0.35	0.10	0.12	0.12	0.10	0.12	0.12
*F*_*model*_	12.20***	28.50***	25.07***	8.60***	8.08***	7.07***		8.08***	7.11***
*Delta R*^*2*^	0.15	0.22	0.01	0.11	0.03	0.01	0.12	0.02	0.01
*F*_*change*_	12.20***	59.03***	1.01	8.60***	6.12**	0.17	7.95***	3.85*	1.25

^a^ Coded as 0 = Office, 1 = Home

^*^ *p* < .05

^**^ *p* < .01

^***^ *p* < .001

In model 2, the interaction between Supportive leadership and Place of work is added to investigate if Place of work moderates the association between Supportive leadership and outcome. The result show that Place of work did not moderate any of the relationships between Supportive leadership and investigated outcomes.

## Discussion

The aim of this study was to examine the place of work as a moderator for the effectiveness of leadership on employee well-being.

The results show that Supportive leadership was related to all investigated well-being variables in the hypothesised directions. Thereby, Hypothesis 1 is supported. Although this study, in line with previous studies, finds significant associations between leadership and the studied outcomes, the explanatory value is quite low for the measure of general well-being, compared with those who measure work-related well-being (i.e., Job satisfaction). It thus seems to be the case that leadership is primarily important for work-related well-being, and to a very small extent for well-being in general. It may seem logical that leadership exercised in a work context is also primarily important for aspects that concern work-related well-being, but it also clarifies the importance of examining multidimensional concepts with different indicators. This is something that has been pointed out in previous literature reviews [5–7], but which has rarely been investigated empirically.

The results also show that the Place of work did not moderate the relationship between Supportive leadership and the investigated outcomes. Thus, Hypothesis 2 is not supported. Instead, the results suggest that the Supportive leadership of managers is equally important, regardless of the workplace of the employee.

Investigations regarding the importance of leadership for employee well-being have mostly been conducted either on on-site workers [2–8] or on employees working from home [36, 39, 40], and these have found significant associations for both groups. Only a few previous studies have investigated whether the effect of leadership differs for these two groups [12–17], with varying results. However, these studies suggest that leadership is equally or more important when working from home compared to when working in the regular office, which was the basis for how the hypothesis was formulated in the current study. In this study, we chose to use three different measures of well-being to capture its multidimensionality. It is conceivable that different aspects of employee well-being are affected in different ways by leadership, as our study suggests. Social isolation has previously been highlighted as a problem for employees working from home [45, 58], and Golden and Veiga [12] suggest that leadership is more important for employees working from home as they have less contact with colleagues. The leadership of the supervisor becomes more important when working from home as office workers have collegial social support to compensate. The findings of this study, however, oppose both of the previous ideas as Supportive leadership is equally important for employees working on-site as it is for employees working from home. Regardless of the workplace, the employees still need their manager to show concern about their welfare and support them in their work.

The current study was, however, conducted during a pandemic where it was recommended by authorities to work from home, which many did. Consequently, also those who remained at their workplaces were more socially isolated than usual, and leadership, therefore, seem to be equally important for both groups, which may be an explanation for the lack of moderation. Furthermore, previous research has also found that managers have tried to compensate for the unusual circumstances of the Covid-19 pandemic by communicating more often with employees than before the pandemic to support them and to ensure the work went as planned [27, 52]. It is therefore a possibility that the lack of differences found in the samples regarding the investigated variables may be the result of managers devoting more time to communication and support than previous regardless of the workplace. Place of work was also measured dichotomously in this study. However, it is conceivable that curvilinear relationships exist, similar to what has been found regarding the relationship between stress and performance [59]. Leadership may thus have a similar effect on employee well-being up to a certain degree of working remotely. But because of how the phenomenon was measured, that cannot be distinguished in the current material. It is also possible that leadership has a different effect within the group that is working from home related to the communication platforms [60] or the severity of the crisis the Covid-19 pandemic was perceived to be [61, 62].

It is clear that more research is needed that investigates the effect of leadership on employee mental but also physical well-being, particularly since the possibilities to work ergonomically may differ between work undertaken from home as compared to on-site in the regular office [63, 64]. In this study, we controlled for the differences in access to Work equipment in order to investigate the effect of leadership. In fact, the only difference found between employees working from home and working from the usual office was in the perception of having suitable Work equipment. However, remote work raises issues about the limits of employers’ responsibility for the work environment, where the legal responsibility may be more encompassing than the practical possibilities for offering adequate support (physical and psychological) when employees are working from home. The pandemic may, as discussed above, have influenced the results where a moderation effect for these issues may occur had remote work not been externally mandated. This study thus clarifies the need to not limit investigations to those who worked from home, or those who worked on-site due to necessary work, but also to examine those who were allowed to work from home but who did not, and how they were affected by such a drastic change brought about by the pandemic.

### Limitations and further research

Some further limitations of the study should be addressed. The first limitation concerns the cross-sectional design, which makes it impossible to distinguish the causal relationship between leadership and well-being. However, previous cross-sectional and longitudinal research investigating the topic has found similar associations in the expected direction [2–8].

A second limitation concerns the risk of common method bias as all scales were rated by the employees [65, 66]. The employee perception of leadership seems relevant to capture, but perhaps the subjecting ratings of well-being should be combined with more objective measures of health.

The third potential limitation concerns the uneven distribution of the sample, particularly the group who decided to mainly work on-site. On the one hand, a larger group would make it easier to detect small differences, and it is possible that a higher power would identify a small moderation effect. On the other hand, people working from the office despite having the opportunity to work from home do not seem to be that prevalent, and enlarging this group would likely increase the likelihood of including bias, such as differences in the ability to perform work tasks at home.

A fourth potential limitation concerns the investigated leadership. While the scales were taken from the well-used and validated COPSOC instrument [55, 56], it has been developed from an occupational health perspective and not from a particular leadership theory. There are likely important leadership behaviours not addressed in the scale. Future research should include leadership perspectives that enable differentiation between leadership behaviours. Stoker et al. [67], for instance, showed that working from home requires reduced managerial control and increased delegation to promote employee productivity, but the rapid pace of the Covid-19 pandemic did not enable the managers the time to make such changes in their leadership. Qualitative research could be used to provide a more nuanced and richer description of specific leader behaviours that are important for well-being when working from home as compared to working on site. Future research should also investigate the importance of the managers’ job location in relation to the well-being of employees who worked on-site or from home. Previous studies have shown impaired job satisfaction when both managers and employees work from home [68].

## Conclusions

This study shows that there are few differences between employees working from home or working on-site during the Covid-19 pandemic, at least regarding perceived leadership and well-being. The leadership of the closest manager seems to be important for well-being regardless of the worksite. Previous research addressing the effects of Covid-19 is often focused on those working from home, or those who had to work from their regular office. This study therefore contributes by investigating those who decided to work in their regular office despite having the opportunity to work from home – a group that has not received a great deal of attention. Furthermore, this study contributes to the scarce research about boundary conditions regarding the relationship between leadership and employee well-being. This study also contributes to research about teleworking by showing that leadership is an important factor in contributing to employee well-being when working at home. If the future work life consists of more remote work as the public debate currently suggests, ensuring the leadership of managers seem to be of utmost importance.

## Data Availability

The dataset for the current study is held within Linköping University and is available from the corresponding author on reasonable request.
